# Coronary Perfusion Following a Transcatheter Aortic Valve Replacement in Either Reanimated Swine or Preserved Human Hearts

**DOI:** 10.31083/RCM40103

**Published:** 2025-09-26

**Authors:** Michael A. Bielecki, Julianne H. Spencer, Paul A. Iaizzo

**Affiliations:** ^1^Visible Heart® Laboratories, Departments of Surgery and Biomedical Engineering and the Institute for Engineering in Medicine, University of Minnesota, Minneapolis, MN 55455, USA; ^2^Structural Heart and Aortic, Medtronic, Mounds View, MN 55112, USA

**Keywords:** pre-clinical research, TAVR, coronary obstruction

## Abstract

**Background:**

Transcatheter aortic valve replacements (TAVRs) have become the predominant solution for treating patients with severe aortic valve stenosis. Meanwhile, procedural coronary obstructions and/or occlusions post-TAVR have subsequently become greater concerns as the use of TAVR increases in younger populations. Therefore, this preclinical study aimed to develop methodologies to assess coronary perfusion following a TAVR in both reanimated swine hearts and preserved human specimens perfused in a pulsatile system.

**Methods:**

This study employed Visible Heart® methodologies to functionally reanimate seven swine hearts. Endoscopic video cameras were used to enable direct visualization of the aortic root throughout these experimental procedures. Pressure wires were placed in the desired coronary arteries, and measurements were collected both before and after the TAVR. Subsequently, these reanimated hearts were scanned using micro-computed tomography (micro-CT), and the valve placements were assessed at resolutions >20 microns. Similar methodologies were utilized to study 13 perfusion-fixed human hearts, using a pulsatile pump, their valves were made functional, and the coronaries were perfused.

**Results:**

Pressure measurements from the left anterior descending arteries (LADs) were normalized to the recorded aortic pressures and the percentage difference from the pre- and post-TAVR and were correlated to the following features: commissural alignments (*p* = 0.274), valve implant depths (*p* = 0.546), left coronary sinus height (*p* = 0.127), left coronary ostium heights (*p* = 0.012), and estimated leaflet to ostium distance (ELOD) (*p* = 0.001).

**Conclusions:**

These studies suggest that there may be stronger correlations between the ELOD and coronary perfusion post-TAVR than pre-procedural measurements of left coronary ostium heights. Left sinus heights, commissural alignments, and implant depths did not correlate significantly relative to coronary perfusions post-TAVR. These results could be further explored in various clinical studies and potentially used to provide additional insights into TAVR procedures across different patient anatomies, informing innovations in device design.

## 1. Introduction

As transcatheter aortic valve replacement (TAVR) has become an established treatment for patients with symptomatic severe aortic valve stenosis (AS) and there has been an increase in interest in the middle to long-term associated complications. Compared to traditional surgery, most concerns about the long-term viability of TAVR center on the prostheses’ durability. As these therapies are extended to younger patients, there has been heightened awareness of how the positioning of the prosthetic valve within the aortic root can influence future coronary access and flow. The topic of commissural alignment has recently been defined as a research area of importance for the ease of cannulation of the coronary arteries post-TAVR and in patients with potential for future valve-in-valve therapies.

Coronary obstruction is expected to remain a rare complication; however, when it does occur, it can be potentially catastrophic. Coronary ostium heights are measured from the pre-procedural CT scan, and have been used to evaluate the risk of coronary obstruction following the TAVR (coronary ostium heights less than 10 mm–12 mm are typically considered at risk for obstruction) [1, 2, 3, 4, 5, 6]. Recently, Oh *et al*. [2] proposed that measuring the expected leaflet to ostium distance (ELOD), which is defined as the shortest distance between the coronary artery ostium and the corresponding expected position of the native leaflet displaced by the transcatheter heart valve (THV), may be an effective predictor of coronary obstruction post-TAVR. In any case, acute obstruction incidence remains low and is generally linked to the implanted THV and anatomical interactions, such as valve leaflet proximity to ostia, displaced native leaflet, depth of implant, and commissural post facing the ostia.

The primary research objective for our team was to develop methodologies to assess coronary perfusion following TAVR and other associated procedures in both reanimated swine and human hearts, as well as in perfused human hearts that have been fixed in a formalin buffer. Procedural steps were observed using endoscopy to provide qualitative insights into valve positioning and its interaction with the aortic root.

## 2. Materials and Methods

The Visible Heart® Laboratories have developed unique methodologies in large mammalian hearts to visualize the interactions of TAVR with the aortic root, and also evaluate how these devices may affect coronary perfusion in the functional cardiac tissue [7]. Two basic research methodologies were utilized to investigate coronary access and perfusion following a given TAVR procedure: reanimation of the heart with a clear perfusate and pulsatile perfusion of hearts fixed in a formaldehyde-based buffer.

### 2.1 Swine Heart Reanimation

**Fig. 1.**
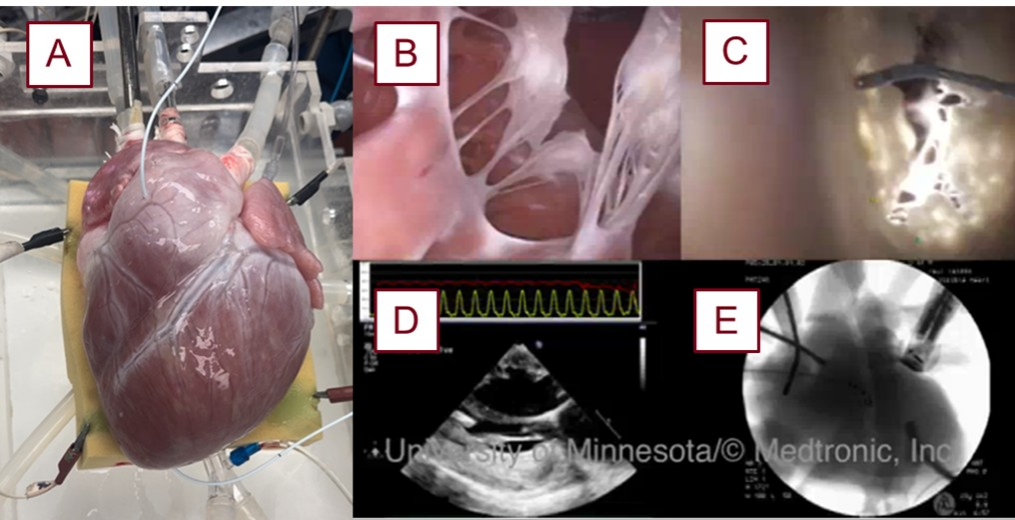
**Image of a swine heart that was reanimated with the various imaging modalities utilized for these experiments**. A reanimated swine heart (A), prepared using the above methodologies [7]. Endoscopic video footage of the mitral valve from the left ventricular apex (B) and from the left atrium (C). Other modes of imaging used in the lab, such as echocardiography (D) and fluoroscopy (E), are used for further insights and device implementation.

For heart reanimation, swine hearts were delivered cardioplegic solution, isolated, cannulated, and then placed onto the Visible Heart® apparatus. The apparatus perfuses the heart with a modified Krebs-Henseleit buffer, providing the necessary ions and nutrients for the heart to contract without stimulation from a pacemaker [7], while remaining transparent for endoscopic visualization of cardiac interventions from within the heart. Swine models are typically selected over ovine or canine models for their greater anatomical and physiological similarities to humans and thus were the primary model used in these experiments (see Fig. 1, Ref. [7]).

**Fig. 2.**
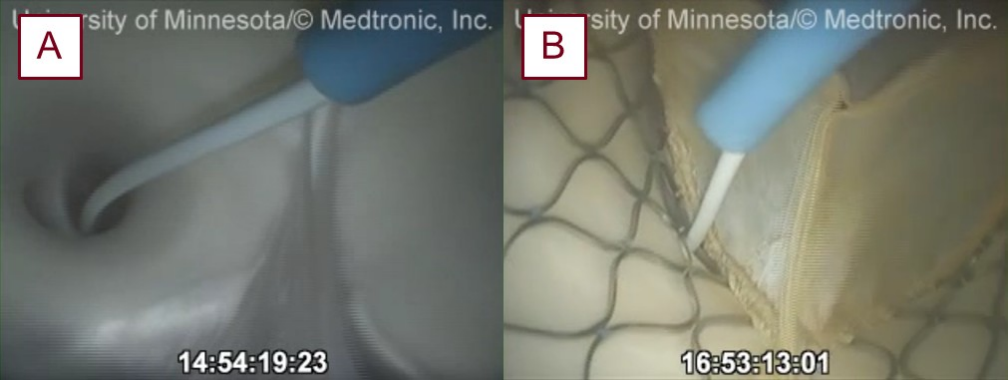
**Endoscopic views of coronary cannulation and delivery of the coronary pressure wire into the LAD before (A) and post-TAVR (B) in a reanimated swine heart**. LAD, left anterior descending artery; TAVR, transcatheter aortic valve replacement.

**Fig. 3.**
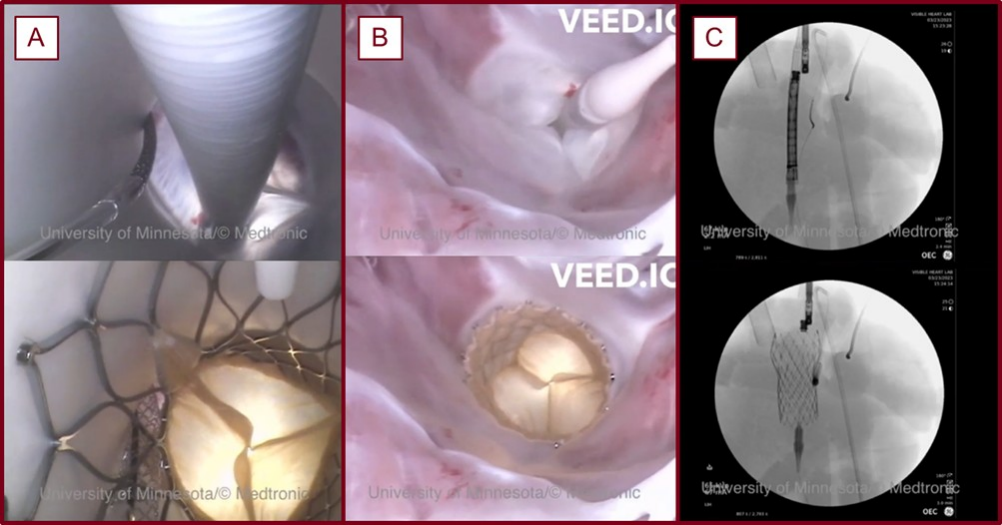
**A TAVR procedure visualized with endoscopic cameras and fluoroscopy conducted in a reanimated swine heart within the Visible Heart® Laboratories [7]**. The aortic view (A), the view from the left ventricular apex (B), and a fluoroscopic view (C) before and after a TAVR procedure with a chimney stent.

Before the deployment of a prosthesis, a Millar pressure catheter (Millar LLC, Houston, TX, USA) is placed into the left anterior descending artery (LAD) at a marked depth (see Fig. 2). Pressures were then recorded in the reanimated heart while it was in a native sinus rhythm. Following these measurements, the catheter was removed from the coronary and pulled from the aorta. The aortic root was then visualized via an echocardiogram and used to select the size of a Medtronic Evolut line (Medtronic, Mounds View, MN, USA) THV. The chosen valve was then loaded and delivered via the right brachiocephalic artery. During such, direct visualizations of the aortic valve were obtained from the left ventricle and the aorta using endoscopic video cameras, and fluoroscopy was used further to visualize the anatomy of the aortic root and coronaries using contrast ejected from a pigtail catheter positioned within the non-coronary aortic cusp. Next, a guidewire was advanced past the aortic valve and into the left ventricle of the heart. The transcatheter valve was then advanced past the native aortic valve and positioned and deployed with guidance from fluoroscopy and imaging from the videoscopes (see Fig. 3, Ref. [7]).

Seven swine hearts were prepared and studied using the aforementioned methodology. In each case, varying implant depths and degrees of commissural alignments were assessed, and how each would affect coronary accesses and perfusions were noted. A pressure wire was guided and placed into the LAD to the same coronary depth, and pressure was recorded post-TAVR. In certain cases where coronary cannulation was not trivial to obtain, the pressure wire was inserted into the LAD via the anterior surface of the heart. Qualitative analyses on the relative coronary access were then collected. All animal handling and treatment were following approved Institutional Animal Care and Use Committee protocols at the University of Minnesota. Previously, similar procedures have, albeit rarely, been conducted in reanimated human hearts within the Visible Heart Laboratories® [8].

### 2.2 Preserved Human Heart Perfusion

The Visible Heart® Laboratories has had a longstanding collaboration with a local organ procurement group (LifeSource, Minneapolis, MN, USA) that allocates human heart donations that were rejected for transplant to be used for research. LifeSource secured consent from donors or their families to use organs for research, and, occasionally, these hearts are viable for reanimation. In these cases, similar procedures as described above can be implemented in human tissue. When the hearts are not suitable for reanimation, they are immediately perfusion-fixed in a 10% formalin buffer in an end-diastolic state. These fixed specimens have been used in conjunction with a Vivitro SuperPump (Vivitro Labs Inc., Victoria, B.C., Canada) for TAVR research.

**Fig. 4.**
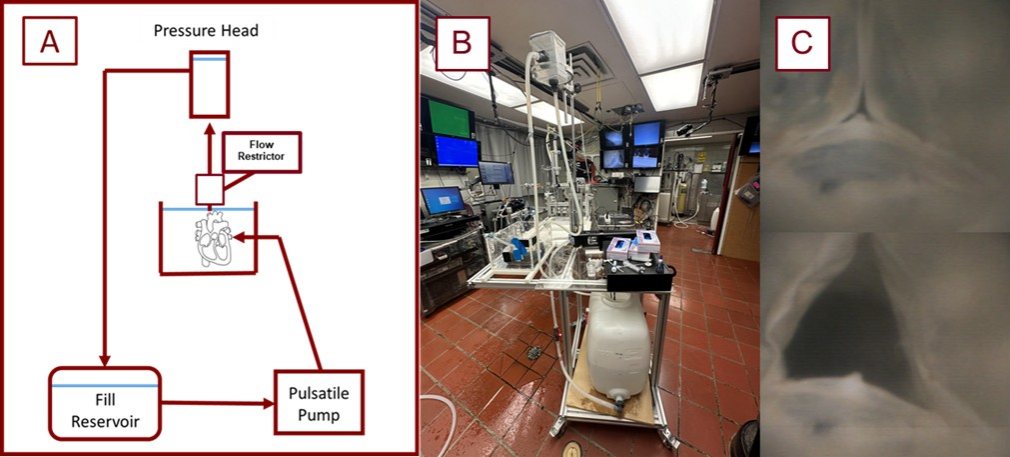
**Schematic and image of the perfusion system utilized in these experiments**. Shown here is a schematic diagram of the employed pulsatile perfusion apparatus used for our experiments (A). The photo (B) shows the pump in use, and the images in column (C) equate to the shut and open states of a THV in a preserved heart being perfused on the system. THV, transcatheter heart valve.

**Fig. 5.**
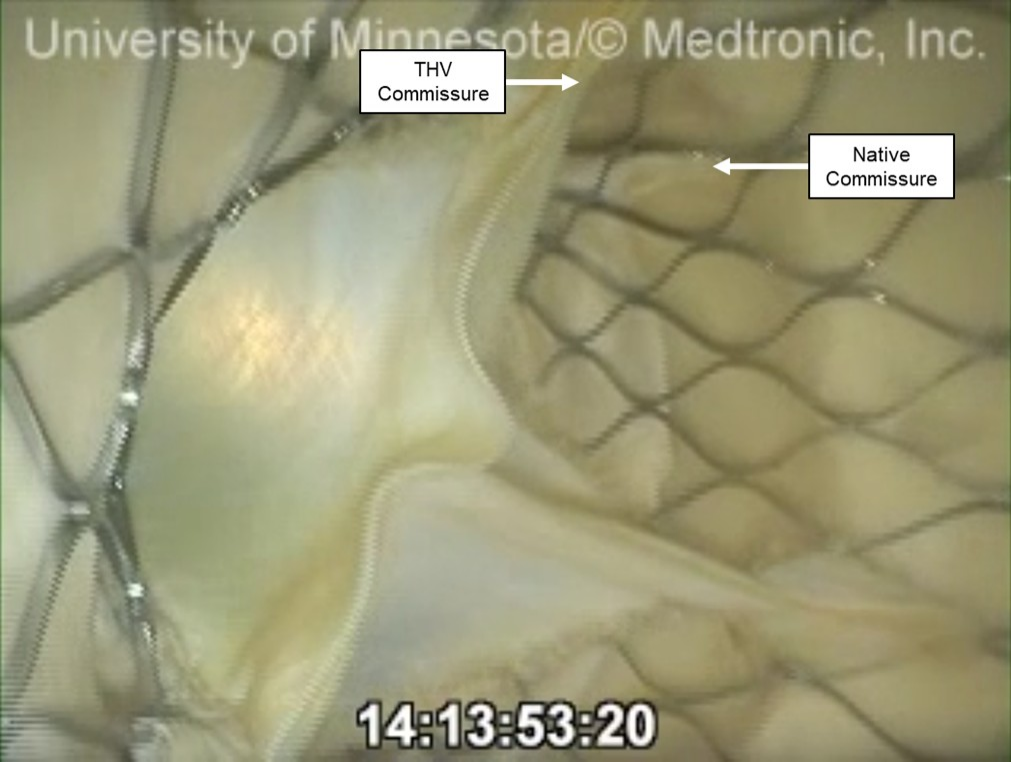
**An endoscopic video view from the aortic root of a preserved human heart that received a TAVR while on the pulsatile perfusion system**. The native commissure and the THV commissures may be considered relatively well aligned in clinical practice, but were not aligned in the scope of this experiment. This left the THV commissure in the 4 o’clock position, aligning near the center of this coronary cusp. However, access and perfusion were not significantly obstructed by the native leaflet or commissural post.

The outlet of the pulsatile pump is cannulated to one of the pulmonary veins of a given fixed heart. The aorta is then cannulated, and fed through a flow regulator to a pressure head that allows sufficient pressure and resistance for the aortic valve to shut, and for the coronary vessels to be appropriately perfused (see Figs. 4,5). Following a similar procedure to the reanimated hearts, the LAD of a given heart was cannulated, and the pressures were monitored while the pulsatile pump was perfusing the specimen. Following the same procedures described above, a THV was deployed across the specimen’s aortic valve. In a sample of n = 13 fixed hearts, the pressures were recorded, the coronary accesses were qualitatively assessed, and these hearts were micro-computed tomography (micro-CT) scanned.

### 2.3 Micro-CT Analysis

**Fig. 6.**
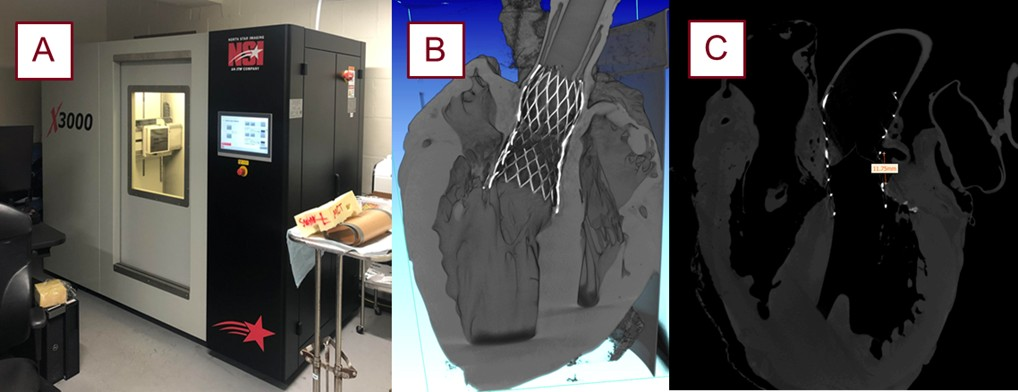
**The micro-CT scanner utilized in these experiments with the generated reconstruction and segmentation software**. The Northstar Imaging X3000 micro-CT scanner (North Star Imaging, Rogers, MN, USA) (A). Scans are reconstructed (B) and transferred to Mimics Materialise (Materialise NV, Leuven, Belgium) for measurements, as exemplified with the coronary height measurement (C). micro-CT, micro-computed tomography.

**Fig. 7.**
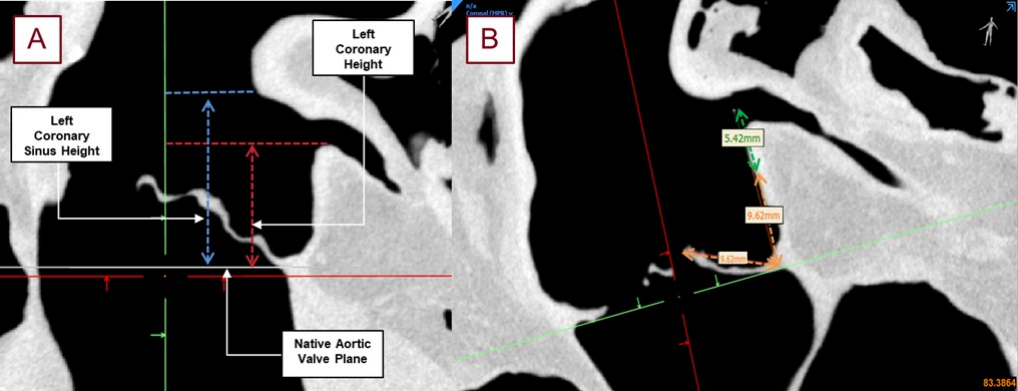
**Shown here are the typical measurements taken from the obtained micro-CT scans without a TAVR placed in the anatomy with roughly 90 micron resolution**. The left coronary ostium height, shown in red, from the base of the coronary cusp to the base of the left coronary ostium (A). The left coronary sinus height, shown in blue, was the measurement from the nadir of the left coronary cusp to the top of the sinus. The left coronary ostium height, shown in red, is a more standard pre-procedural measurement for assessing coronary obstruction. The estimated leaflet to ostium distance (ELOD) (B) was also determined to compare its correlation with coronary obstruction [2]. The orange measurement shows the length of the leaflet associated with the desired coronary cusp. This distance was projected, perpendicular to the aortic valve plane, and the distance from the center of the coronary ostium to this leaflet length was recorded as ELOD, shown in green.

**Fig. 8.**
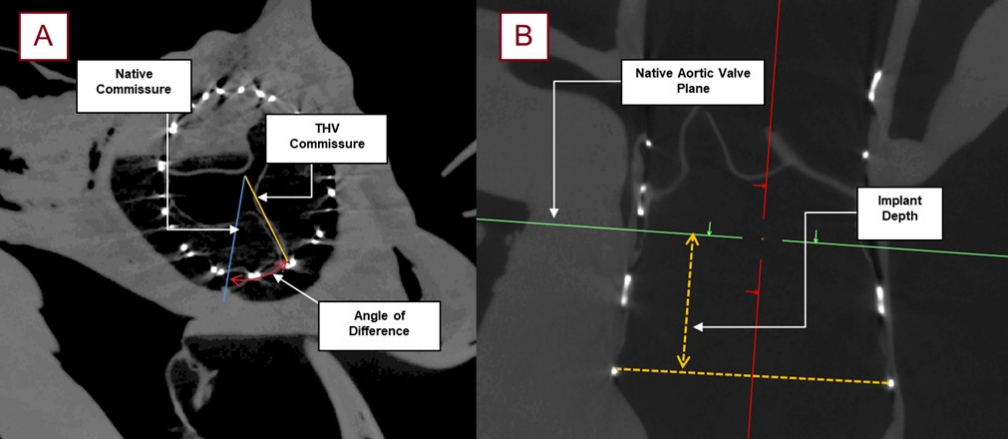
**Images obtained from micro-CT scanning following a TAVR procedure with roughly 90 micron resolution; this allowed for the post-TAVR measurements utilized in this study**. The relative angle (red) between the native commissure (blue) and the commissural post of the THV (yellow) was collected for each specimen (A). Additionally, the subsequent implant depth (yellow) was measured from the lowest attachment of the left coronary cusp, to the base of the THV frame (B).

Following these interventions, the given heart was removed from the apparatus or perfusion system and imaged using a North Star Imaging X3000 micro-CT scanner (North Star Imaging, Rogers, MN, USA) (see Fig. 6). Each scanning dataset (with roughly 20 micron resolution) was reconstructed and then transferred to Materialise Mimics (Materialise NV, Leuven, Belgium) software for subsequent computational modeling and analysis. Measurements regarding both the prosthesis and anatomical features of the aortic root included: left coronary artery ostium height, coronary sinus height, commissural alignment, and implant depth. Implant depth and coronary height measurements were taken from a plane created at the nadirs of the native aortic valve leaflet attachments, while the position of the THV commissural posts was measured in degrees relative to the native commissures, using the center of the aortic root as the reference point (see Fig. 7, Ref. [2]; Fig. 8).

### 2.4 Coronary Pressure Analysis

**Fig. 9.**
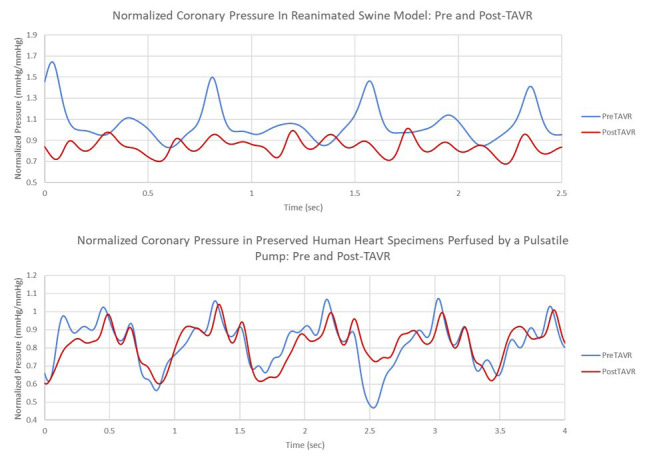
**Examples of the pressure data collected in both a reanimated swine (Top) heart and the pulsatile perfusion of a preserved human heart specimen (Bottom)**.

Continuous pressure measurements from the LAD and the aorta were collected during these experiments. To normalize the pressure data, the LAD pressure was divided by the corresponding aortic pressure for each sample point (see Fig. 9). An average from five of the peak normalized pressures from both the pre- and post-TAVR timepoints were calculated, and the percent reduction of the average normalized pressure from before and after the prosthesis was recorded for each case and used in correlation with the various anatomical features, and device placements assessed in these experiments.

### 2.5 Statistical Analysis

For each of the measurements collected (see Table 1), Spearman’s correlations (*r*_s_) were collected, and a two-tailed *t*-test was used to examine the relative significance of the findings (see Table 2). Additionally, the left coronary ostium height and ELOD groups were subjected to a non-linear regression with a logarithmic model, and a standard deviation was determined for each of the models.

**Table 1.** **Measurement averages of the experimental specimens**.

	Avg commissure to native commissure angle (degrees)	Avg left coronary sinus height (mm)	Avg implant depth (mm)	Avg left coronary ostium height (mm)	Avg ELOD (mm)
Preserved human	24.506	16.360	4.273	11.641	3.328
Swine	17.701	13.210	6.970	7.199	2.841
Cumulative	21.104	14.785	5.622	9.420	3.085

All values represent the mean anatomical and procedural measurements collected following a TAVR procedure. These measurements were collected from micro-CT scans of the swine and human heart specimens.

**Table 2.** **Anatomical and procedural measurement data with correlation to coronary pressure change post-TAVR**.

	Average	Range	Median	P25	P75	Std deviation	Spearman correlation	*p* value
Commissural angles (Degrees)	24.506	54.250	18.140	9.538	34.333	16.477	0.257	0.274
Left coronary sinus height (mm)	16.360	11.580	15.630	13.230	16.988	2.876	0.353	0.127
Implant depth (mm)	4.273	18.090	5.405	3.535	7.738	4.309	0.053	0.546
Left coronary artery ostium height (mm)	11.641	9.080	10.230	8.030	11.538	2.421	0.548	0.012
ELOD (mm)	3.328	6.180	2.750	1.928	3.813	1.646	0.702	0.001

These anatomical and procedural measurements were obtained following a TAVR procedure and were correlated with the changes of coronary pressures following the deployment of the valves. Median, quartiles, and standard deviations are reported to describe the distributions. Spearmen correlation coefficients were calculated to assess the relationships between each variable and the observed changes in coronary pressures.

## 3. Results

The developed experimental setups were successfully able to monitor the coronary pressures before and after a TAVR procedure. Both the reanimated swine hearts and the perfused human heart specimens were successfully used in these TAVR procedures, and endoscopic footage was collected within each specimen. Multimodal imaging was successful in evaluating clinical deployment techniques, and the video imaging enhanced the understanding of the final implant position and its interactions with the aortic root and surrounding tissues. The preserved hearts showed similar performances to the reanimated functional swine regarding what would be expected relative to coronary pressures. The pulsatile waveforms in the perfused hearts sufficiently mimic that which were produced by the reanimated hearts and were also similarly sensitive to obstructions.

Commissural alignment based on the angle between the native and THV commissure (*r*_s_ = 0.257, *p* value = 0.274), left coronary sinus height (*r*_s_ = 0.353, *p* value = 0.127), and implant depth (*r*_s_ = 0.053, *p* value = 0.546) seemed to have negligible correlations and significances relative to changes in coronary pressures (see Tables 1,2).

**Fig. 10.**
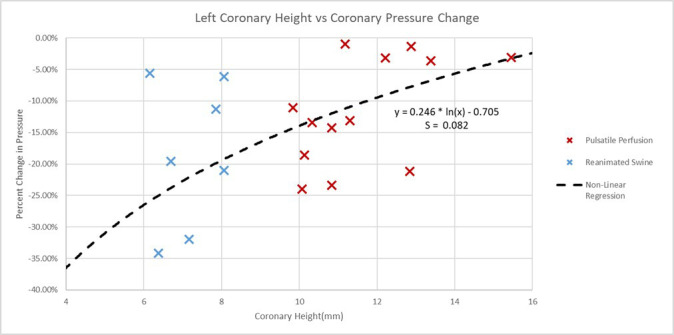
**Left coronary artery ostium height vs percent coronary pressure change plot**. Plotted here are the heights of the left coronary ostium from the aortic plane, which were measured for both the swine and preserved human specimens. The pressures in the specimen’s coronary arteries, either during reanimation or pulsatile perfusion, were normalized to the simultaneous aortic pressures. These normalized pressure values were taken pre- and post-TAVR procedures and plotted as the percent differences. A determined non-linear regression showed a standard deviation (S) of 0.082 to the logarithmic model, and a *p* value of 0.012.

One tool to assess the risk of coronary obstruction would be the ostium height from the aortic annulus. The coronary heights in these experiments elicited greater correlations and significance (*r*_s_ = 0.548, *p* value = 0.012) than commissural alignments, implant depths, and sinus heights (see Fig. 10).

**Fig. 11.**
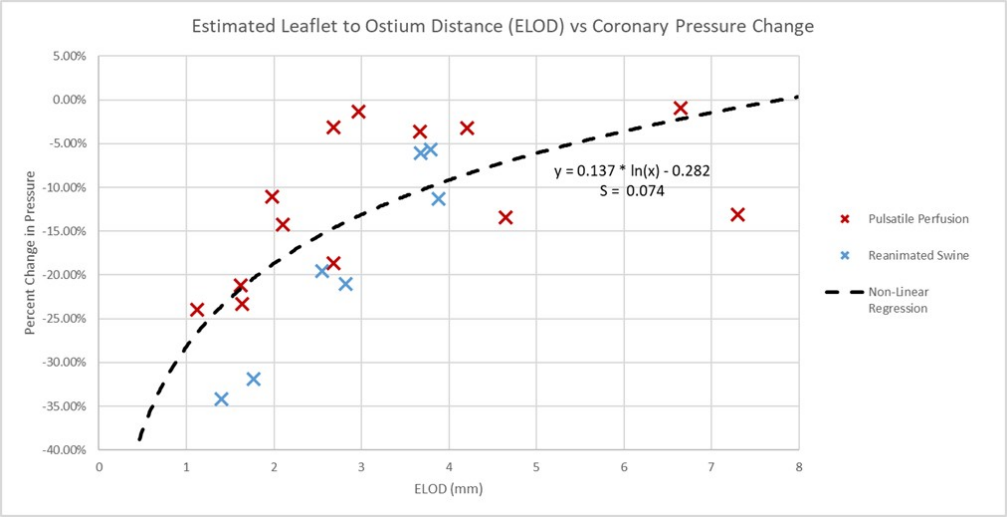
**Estimated leaflet to ostium distance vs percent change in coronary pressure plot**. Plotted here are the estimated leaflet to coronary ostium distances (ELOD), obtained from the micro-CT scans, recorded for each of the specimens, and correlated to the percent changes in normalized coronary pressures. This associated non-linear regression had a smaller standard deviation (S) of 0.074 to the logarithmic model than the left coronary ostium height, and a stronger significance with a *p* value of 0.001.

The ELOD measurements were proposed as more accurate predictors for potential coronary obstructions [2]. The distances from the leaflets to the outer diameters were also collected for these specimens following these procedures and then compared to the percent changes in normalized coronary pressures. The average estimated leaflet to ostium distances (ELOD) for the preserved human and swine specimens were 3.329 mm and 2.841 mm, respectively (see Fig. 11). The variances in the associated logistic regression were less than what was seen in the left coronary artery ostium height model and elicited a higher correlation and significance (*r*_s_ = 0.702, *p* value = 0.001).

## 4. Discussion

There is a significant amount of literature exploring coronary obstruction post-TAVR. Such complications of TAVR are generally defined as where the valve frame, or leaflets, either partially or completely obstruct the flow of blood to one or both coronary ostia. Currently, coronary obstructions have been observed in 0.7% [6, 9, 10, 11] of TAVR interventions, where they have required either rescue percutaneous coronary intervention (PCI) or emergency coronary artery bypass. Furthermore, access to the coronaries following a TAVR intervention for future required PCI procedures can also be hindered by the previously placed prosthesis. In other words, as TAVR is increasingly utilized in lower-risk patients with higher long-term prognoses, the concern for access for intervening in coronary artery disease (CAD) with PCI will also increase.

The average left coronary artery ostium height in the studied preserved human specimens was 11.64 mm, and 7.20 mm in the reanimated swine specimens. Generally, coronary ostia heights below 10–12 mm are believed to be at risk of coronary obstruction [2, 4, 5, 6, 11, 12, 13]. The preserved human specimens were slightly lower than the average left coronary ostium height [14] and the swine specimens were shown to have much lower coronary ostium heights, indicating that these experiments were interesting challenge cases for assessing coronary obstruction post-TAVR. In addition, the suggested cutoff value for ELOD was 2 mm [2]. Five of the specimens from both groups were found beneath this cutoff value, and correspondingly, these specimens all experienced greater than 20% reduction in coronary pressure.

**Fig. 12.**
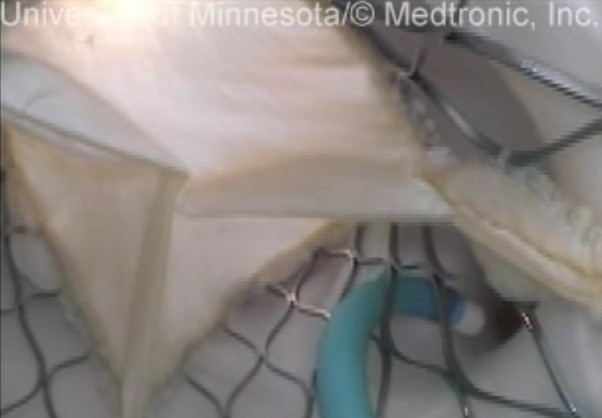
**Cannulation of a coronary ostium through the frame of a self-expanding transcatheter heart valve with poor commissural alignment**. A TAVR intervention was conducted in a reanimated swine heart where the commissural post of the prosthesis was located in the center of the native coronary cusp. While the post was not aligned with the commissures of the native valve, the coronaries were still accessible in this case, as the guide catheter could still pass through the frame and navigate around the post. Furthermore, flow to the coronaries does not seem to be significantly obstructed by the commissural post.

These preliminary results from these preclinical studies provide support for the assessments of ELOD in the preprocedural measurements for TAVR as a method to reduce the incidence of reduced coronary perfusion. This described method highlighted a need for considering additional factors such as the length of the valvular leaflet and relation to coronary height, rather than coronary ostium height alone. While we described here that there was an observable correlation between coronary height assessments and resultant coronary pressures post-TAVR (this is traditionally the quicker and more commonly used predictor for coronary obstructions), we also determined that the ELOD was also a significantly correlated predictor for potential coronary obstructions and was considered to be more correlated than measuring the coronary ostium height alone. This was a consistent finding utilizing both reanimated swine hearts and pulsatile perfused formalin-fixed human hearts for these assessments. Additionally, mild, moderate, and severe misalignment of the native and THV commissures were not well correlated with a decrease in coronary perfusion post-TAVR in our investigations (see Fig. 12).

More recently, a standardized tool for coronary obstruction prediction is the virtual transcatheter aortic valve replacement to coronary (VTC) distance, where a virtual valve is digitally inserted into the preprocedural scan, and the distance from the most proximal opening of the coronary to this virtual valve is recorded [2, 15, 16, 17]. This technology was not available to the team, and such measurements were not collected.

While measurements like the coronary height and VTC are well defined in literature for their association with potential post-TAVR coronary obstruction, a direct correlation to invasively measured coronary pressures is not well defined. The methodologies conducted in these experiments create an interesting preclinical model for post-TAVR coronary obstruction that can further illuminate how these anatomical and procedural features affect coronary perfusion.

This study may have several limitations that warrant consideration. First, the study of small numbers of swine and human hearts reduced the statistical power, perhaps making the procedural findings of this research less conclusive. Future retrospective clinical studies are needed to better understand how anatomical features and device placements may contribute to coronary obstructions. Second, while swine cardiac anatomy has historically served as a strong surrogate for human anatomies [18, 19, 20], it does not fully replicate the coronary ostial anatomies of human hearts. Specifically, the coronary ostial heights in swine specimens are generally much lower than in humans [21, 22], and the swine left main arteries are significantly shorter. Additionally, the curved distance of the leaflet from the aortic valve plane can be challenging to accurately collect consistently, and such a linear measurement was collected for the purpose of this study. Furthermore, this study was conducted with self-expanding valves, and not with balloon expandable valves, and coronary pressure changes would likely vary between these two valve types. The use of preserved human hearts introduces its own limitations, as the preservation processes can make tissues stiffer, and less elastically responsive than living tissue. Yet, this may not be considered as significant of a factor for highly calcified tissues. The pulsatile pump employed in the study simulates flow from the left ventricle but does not fully replicate the patient’s full aortic flow waveforms. Despite these limitations in both swine and preserved human tissues, the normalization processes and individual assessments of pressure reductions showed to be comparably responsive in both models, supporting the overall relevance of our findings.

## 5. Conclusions

The described methodologies developed by the Visible Heart® Laboratories provide unique pre-clinical research tools for assessing a given TAVR procedure and subsequent coronary access and perfusion. These direct visualizations and pressure quantifications of post-TAVR coronary flows provide a preclinical framework for identifying anatomical and procedural factors that may result in coronary obstructions. This work highlights the need for a larger study that utilizes similar models to better understand the effects of TAVR design, cardiac anatomy, and valve delivery on coronary perfusion.

## Availability of Data and Materials

The anatomical data, video files, and other imaging results are publicly available upon request. Other functional anatomical footage of the heart can be found on the atlas of human cardiac anatomy website: https://www.vhlab.umn.edu/atlas/.
